# Regulatory Features for Odorant Receptor Genes in the Mouse Genome

**DOI:** 10.3389/fgene.2017.00019

**Published:** 2017-02-21

**Authors:** Andrea Degl’Innocenti, Anna D’Errico

**Affiliations:** ^1^Max Planck Institute of BiophysicsFrankfurt am Main, Germany; ^2^Cell and Developmental Biology Unit, Department of Biology, University of PisaPisa, Italy; ^3^Center for Micro-BioRobotics, Italian Institute of Technology, Sant’Anna School of Advanced StudiesPisa, Italy

**Keywords:** allelic exclusion, element, enhancer, epigenetics, gene expression, odorant receptor gene choice, promoter, TFBS

## Abstract

The odorant receptor genes, seven transmembrane receptor genes constituting the vastest mammalian gene multifamily, are expressed monogenically and monoallelicaly in each sensory neuron in the olfactory epithelium. This characteristic, often referred to as the *one neuron–one receptor* rule, is driven by mostly uncharacterized molecular dynamics, generally named *odorant receptor gene choice*. Much attention has been paid by the scientific community to the identification of sequences regulating the expression of odorant receptor genes within their *loci*, where related genes are usually arranged in genomic clusters. A number of studies identified transcription factor binding sites on odorant receptor promoter sequences. Similar binding sites were also found on a number of enhancers that regulate *in cis* their transcription, but have been proposed to form interchromosomal networks. Odorant receptor gene choice seems to occur via the local removal of strongly repressive epigenetic markings, put in place during the maturation of the sensory neuron on each odorant receptor *locus*. Here we review the fast-changing state of art for the study of regulatory features for odorant receptor genes.

## Introduction

Many animals rely strongly on olfaction in order to get information about their surroundings, look for food, escape from predators, find a mate and communicate with each other. The transcriptional regulation of odorant receptor genes – comprising in mice ∼ 1100 intact members (Buck and Axel, 1991; Niimura et al., 2014) – displays an almost unique feature: from the whole set, a single odorant receptor gene is monoallelicaly expressed in a single sensory neuron. This singularity is driven by mostly uncharacterized molecular dynamics, collectively termed *odorant receptor gene choice*, which seem to occur via local removal of strongly repressive epigenetic marks put in place on each odorant receptor *locus* during the maturation of the sensory neuron.

The scientific community has tried to identify sequences that regulate the expression of odorant receptor genes within their *loci*, which normally contain groups of related genes tightly arranged in genomic clusters (Sullivan et al., 1996; Niimura et al., 2014). Several studies identified transcription factor binding sites (TFBSs) on odorant receptor promoter sequences. Similar binding sites were also found on a number of enhancers located in proximity to odorant receptor genes. Those enhancers regulate *in cis* their transcription, and seem to form interchromosomal networks.

A solid amount of evidences shows that odorant receptors are not only involved in odor detection (Buck and Axel, 1991), but also in neuronal maturation (Lyons et al., 2013), axonal sorting (Mombaerts et al., 1996; Wang et al., 1998; Vassalli et al., 2002) and neuronal longevity (Santoro and Dulac, 2012). However, their peculiar gene expression and how it is achieved still represent a fundamental open question.

## The Mouse Olfactory System

In mouse the task of sensing a vast range of molecules is run by the main and the accessory olfactory systems. The main olfactory system includes the main olfactory epithelium (MOE), which lines the turbinates in the posterior nasal cavity, and the main olfactory bulb (MOB) in the brain. The MOE is a neurogenic pseudostratified epithelium, which houses basal cells, supporting cells, Bowman’s glands, and olfactory sensory neurons (OSNs); these are responsible for the detection of odorants and other ethologically important molecules. OSNs are bipolar neurons with an apical dendrite ending in a knob from which specialized cilia protrude into the mucus of the nasal cavity (Mendoza, 1993; for detailed reviews see also Breer et al., 2006; Tirindelli et al., 2009).

Primary transduction of odors takes place in the cilia, where the chemosensory receptors, either odorant (ORs) or trace amine-associated receptors (TAARs) are expressed (Buck and Axel, 1991; Liberles and Buck, 2006). These are G protein-coupled receptors (GPCRs) whose signals activate the transduction cascade and influence epigenetic gene regulation. ORs can be phylogenetically divided in two subfamilies: class I, comprising ∼125 fish-like intact OR genes, and class II, including ∼1000 intact OR genes specific for mammals (Niimura et al., 2014). OSNs express monogenically and monoallelicaly a single OR gene from the whole genomic repertoire (Ngai et al., 1993a,b; Chess et al., 1994), a feature known as *one neuron–one receptor* rule. However, as recently reported by Greer et al. (2016), a subset of OSNs localized in the recesses of the olfactory epithelium seems to escape this general rule: each OSNs of the *necklace* subsystem expresses multiple MS4As genes, coding for four-transmembrane chemoreceptors; through a yet unknown signaling path, they are mainly involved in detection of pheromones and others ethologically relevant ligands.

Axons from OSNs expressing the same OR gene, after crossing the cribriform plate, bundle together converging on the same location, referred to as a *glomeruli*, in a few stereotypic domains of the MOB (Le Gros Clark and Turner Warwick, 1946; Ressler et al., 1994; Vassar et al., 1994; Mombaerts et al., 1996). Axonal wiring is a process in which the sensory receptor itself has a fundamental role (Mombaerts et al., 1996; Wang et al., 1998; Vassalli et al., 2002; Movahedi et al., 2016).

The accessory olfactory system includes the vomeronasal organ of Jacobson and its projections to the accessory olfactory bulb, located in a posterior dorsal region of the MOB (McCotter, 1912; Breer et al., 2006), and other olfactory compartments: the septal organ of Masera, sited close to the nasal septum, that sends axonal projections to a subset of glomeruli in the MOB; the Grueneberg ganglion, in the anterodorsal region of the nasal cavity, that sends projections to a subpopulation of the necklace glomeruli in the MOB. Neurons found in septal organ and Grueneberg ganglion epithelia are generally called OSNs, although some of those, as well as the already mentioned necklace OSNs, display some peculiarities (Fleischer and Breer, 2010; Greer et al., 2016).

The vomeronasal organ is a blind tubular structure located at the base of the nasal septum and mainly deputed to pheromone detection. It presents a non-sensory region and a sensory pseudostratified epithelium hosting vomeronasal sensory neurons (VSNs), basal stem cells, and supporting cells (Halpern, 1987; Døving and Trotier, 1998; Ishii and Mombaerts, 2008). VSNs are bipolar neurons with a single dendrite ending in a knob that exposes microvilli to the vomeronasal lumen. They are divided in two main subpopulations distributed on an apical and a basal layer and having as receptors members of two different families of vomeronasal GPCRs. Apical VSNs coexpress G-protein subunit G_αi2_ and receptor genes of the family V1R, which includes ∼150 intact genes (out of 300 genes) divided in 12 clades (Dulac and Axel, 1995; Jia and Halpern, 1996). Genes belonging to the same subfamily are organized in clusters (Herrada and Dulac, 1997; Matsunami and Buck, 1997; Ryba and Tirindelli, 1997; Rodriguez et al., 2002; Zhang et al., 2004) and they have monogenic and monoallelic expression (Dulac and Axel, 1995; Rodriguez et al., 1999; Roppolo et al., 2007). Basal VSNs coexpress G-protein subunit G_αo_ and receptor genes of the family V2R, which includes ∼120 intact genes (out of 280) divided in the subfamilies A, B, and D, comprising most of the intact V2Rs gene repertoire, and the subfamily C (seven genes). V2R sensory neurons express a single, apparently stochastically chosen, member of subfamily C plus one or more selected member of subfamilies A, B, or D (Herrada and Dulac, 1997; Matsunami and Buck, 1997; Ryba and Tirindelli, 1997; Martini et al., 2001; Silvotti et al., 2007; Ishii and Mombaerts, 2011). A subset of these neurons may also express non-classical major histocompatibility complex (MHC) *1b H2-Mv* genes (Ishii and Mombaerts, 2008; Leinders-Zufall et al., 2009). Although most of the regulatory features of V1R and V2R genes are still not well known, Enomoto et al. (2011) reported that transcription factor bcl11b has an important role in regulating the fate choice between the V1R and V2R types of VSNs.

A small subset of VSNs, mostly in the apical neurons, monogenically expresses genes coding for formyl peptide receptors (FPRs), GPCRs that are mainly involved in microbial and viral peptide detection (Rivière et al., 2009; Bufe et al., 2015).

## Genomic Organization of Odorant Receptor Genes and Olfactory Coding

Olfactory information is encoded by thousands OSNs, each of which can bind different molecules with different affinity in a combinatorial fashion (Nara et al., 2011; Jiang et al., 2015) that amplifies the odorant discrimination possibilities of the already huge OR repertoire.

The OR gene family is spread across all genome: class I OR genes are in a single cluster on chromosome 7; class II OR genes are scattered on all chromosomes except the 18 and Y, and arranged in clusters distributed in ∼ 50 *loci*, which have a usual intergenic distance of 19–45 kb (Young et al., 2002; Zhang et al., 2007; Clowney et al., 2012), and few more *solitary* genes distant more than 1Mb upstream and downstream from the start and end of their transcripts (Zhang and Firestein, 2002; Godfrey et al., 2004; Malnic et al., 2004; Degl’Innocenti et al., 2016). OR genes are encoded by single exon ∼ 1 kb long and present conserved amino acid motifs characteristic of their family (Lane et al., 2001; Ibarra-Soria et al., 2014; Kanageswaran et al., 2015; Saraiva et al., 2015). As said, the single OR allele expressed in a single OSN determines also its identity, and influence the OSN’s axonal wiring to specific glomeruli in the bulb, resulting in a stereotyped sensory map that depends from not yet known information provided by the OR. Knowing how odorant receptor gene choice works is therefore pivotal to understand also the logic behind the olfactory input integration.

To explain OR gene choice, several evidences point towards molecular mechanisms that lead to the random choice of only one among several OR promoters, possibly through epigenetic dynamics (Chess et al., 1994; Lomvardas et al., 2006; Clowney et al., 2012). Instead, the possibility of gene rearrangements for OR *loci* in the OSN lineage has been excluded, at least for the *locus* of model OR gene *M71*. In fact, cloning a mouse from the nucleus of an *M71*-expressing OSN resets OR gene choice in favor of *M71*, and results in specimens with a normal OR gene expression (Eggan et al., 2004; Li et al., 2004).

## *Cis-*Regulating Sequences for Odorant Receptor Genes

### Odorant Receptor Gene Promoters

OR gene promoters are AT-rich sequences usually lacking a TATA-box, although some do have one (Clowney et al., 2011; Young et al., 2011; Plessy et al., 2012). When present, however, their positions do not closely correlate with the transcription start site of the gene. For many OR genes, initiation of transcription may adhere to the so-called *rule of genomic contrast*: mRNA polymerization would be caused not by specific increase in AT content but by a sudden local variation of it (cf. Clowney et al., 2011). OR promoters typically feature TFBSs for homeodomain and for olfactory/early B transcription factors (Wang et al., 1997; Vassalli et al., 2002; Young et al., 2011; Plessy et al., 2012), whose presence was in some cases confirmed *in vivo* (Rothman et al., 2005; Vassalli et al., 2011). Along with them, other TFBSs were found on their sequences, e.g., for MEF2A (Plessy et al., 2012). TFBSs are considered major players in defining *zonality* of OR gene expression: OSNs found within a given *zone*, i.e., MOE-subdomain with typical transcriptome, choose stochastically their OR allele out of a subset of the whole genomic repertoire. Non-chosen OR promoters are epigenetically silenced by H3K9me3 and H4K20me3 marks (Magklara et al., 2011). From functional studies, minimal promoters appear to be quite short (∼300 bp; Vassalli et al., 2011), and sequences of similar length have proven to be capable to drive punctate, stochastic expression of OR transgenes in the MOE (Vassalli et al., 2002, 2011; Rothman et al., 2005).

### Odorant Receptor Elements

Elements for OR genes are non-genic regulatory sequences traditionally classified as enhancers, although their very nature as facilitators of transcription is debated: it has been proposed that elements differ from typical enhancers in the sense that they control the probability of a given OR gene to be chosen, rather than merely increasing the amount of transcript per cell for all the genes they regulate (Khan et al., 2011; Vassalli et al., 2011). Elements are invariably found within, or in proximity to, OR *loci* (Khan et al., 2011; Markenscoff-Papadimitriou et al., 2014). Their sequences contain, similarly to OR promoters, homeodomain and olfactory/early B TFBSs, plus additional TFBSs like those for Foxj2, Cdx, C/EBPgamma, Bptf (Markenscoff-Papadimitriou et al., 2014). To date, a total of 14 enhancers are though to regulate OR gene expression in the mouse, three of them being robustly confirmed *in vivo*; these are called H, P, and Lipsi (Nishizumi et al., 2007; Bozza et al., 2009; Khan et al., 2011; Markenscoff-Papadimitriou et al., 2014). It was realized long ago that elements might have been somehow involved in OR gene choice (Serizawa et al., 2000, 2003; Lewcock and Reed, 2004; Shykind et al., 2004), but no clear mechanism has been found yet: elements regulate the expression of OR genes in their *in cis* proximities, although Markenscoff-Papadimitriou et al. (2014) has suggested they may possess *in trans* activity with high degree of redundancy. **Table 1** summarizes DNA regulatory features for OR genes.

**Table 1:** DNA regulatory features for odorant receptor genes.

Regulatory feature	Location	Reference
Enriched presence of 8-oxodG (oxidative damage)^a^	Chosen OR allele	Lyons et al., 2013.
Enrichment for DNase I hypersensitive sites	H and P elements (also in OSNs not expressing an OR gene found *in cis* with them), other elements	Markenscoff-Papadimitriou et al., 2014.
Hist2h2be (H2BE) replication-independent histone variant	Position (e) among HeB-encoding genes, histone cluster 2	Santoro and Dulac, 2012.
H3K27ac epigenetic marks	H and P elements (also in OSNs not expressing an OR gene found *in cis* with them), other elements	Markenscoff-Papadimitriou et al., 2014.
H3K27me3 epigenetic marks	Silenced OR *loci*, element-flanking regions	Magklara et al., 2011; Armelin-Correa et al., 2014a,b; Markenscoff-Papadimitriou et al., 2014.
H3K4me1 epigenetic marks	H and P elements (also in OSNs not expressing an OR gene found *in cis* with them)	Colquitt et al., 2014; Markenscoff-Papadimitriou et al., 2014.
H3K4me3 epigenetic marks	Chosen/euchromatic OR alleles	Magklara et al., 2011; Clowney et al., 2012; Armelin-Correa et al., 2014a; Tian et al., 2016.
H3K79me3 epigenetic marks	Element-flanking regions	Markenscoff-Papadimitriou et al., 2014.
H3K9me3 epigenetic marks	Silenced OR *loci*	Clowney et al., 2011; Magklara et al., 2011; Clowney et al., 2012; Lyons et al., 2013; Armelin-Correa et al., 2014a.
H4K20me3 epigenetic marks	Silenced OR *loci*	Clowney et al., 2011; Magklara et al., 2011; Clowney et al., 2012; Armelin-Correa et al., 2014a.
Homeodomain binding sites	Elements	Nishizumi et al., 2007; Bozza et al., 2009; Vassalli et al., 2011; Markenscoff-Papadimitriou et al., 2014.
Homeodomain binding sites	OR promoters	Vassalli et al., 2002, 2011; Hoppe et al., 2003, 2006; Hirota and Mombaerts, 2004; Rothman et al., 2005; Hirota et al., 2007; Clowney et al., 2011; Young et al., 2011; Plessy et al., 2012; Degl’Innocenti et al., 2016; Zhang et al., 2016.
Local variation in AT content associated with transcription start site	OR promoters	Clowney et al., 2011; Magklara et al., 2011.
O/E binding sites	Elements	Nishizumi et al., 2007; Bozza et al., 2009; Vassalli et al., 2011; Markenscoff-Papadimitriou et al., 2014
O/E binding sites	OR promoters	Wang et al., 1997; Vassalli et al., 2002, 2011; Hoppe et al., 2003, 2006; Hirota and Mombaerts, 2004; Rothman et al., 2005; Michaloski et al., 2006; Clowney et al., 2011; Michaloski et al., 2011; Young et al., 2011; Plessy et al., 2012.
Other transcription factor binding sites (Atf5, Bptf, Cdx, C/EBPgamma, Foxj2)	Elements	Markenscoff-Papadimitriou et al., 2014.
Other transcription factor binding sites (MEF2A, TBP, and transcriptional repressors resembling RP58)	(some) OR promoters	Clowney et al., 2011; Michaloski et al., 2011; Young et al., 2011; Plessy et al., 2012


*Summary of regulatory features, either epigenetic or on primary sequences, found in genomic regions regulating OR gene expression with variable degree of evidence (we report specific references for each of them). ^a^While not properly a regulatory feature, enriched presence of 8-oxodG on chosen OR allele is reported for convenience.*

## Odorant Receptor Gene Choice: Repressive Mechanisms

The organization of the nucleus in OSNs plays a role in the regulation of OR gene expression. Instead of being at the nuclear periphery, as in typical eukaryotic cells, constitutive heterochromatin is mainly located in central nuclear region (Solovei et al., 2009; Clowney et al., 2012; Armelin-Correa et al., 2014a). Indeed, in the early differentiation steps of OSNs, long before OR gene choice takes place, robust silencing and packing occurs on OR *loci*. Cytogenetically OR gene *loci* (and their enhancers) become aggregated in a small number of nuclear locations including arrangements named *foci*, tridimensional chromatin structures characterized by the repressive epigenetic marks H3K9me3 and H4K20me3, typical of constitutive pericentromeric and subtelomeric chromatin (Magklara et al., 2011). These marks will be removed later on from a single OR allele, ensuring monogenic and monoallelic expression (Clowney et al., 2012).

Not all OR gene *loci* are confined in *foci*: constitutive heterochromatin is surrounded by more dynamic facultative heterochromatin, and alleles of the same OR tend to reside on different compartments, one within constitutive heterochromatin and the other in facultative heterochromatin. In fact, as for any monoallelicaly expressed gene family, homologous alleles of OR genes are replicated asynchronously (Chess et al., 1994). Consistent with these observations, immunofluorescence staining of the olfactory epithelium for H3K27me3 – a mark for facultative heterochromatin – indicates that it is present in the nuclei of OSNs (Armelin-Correa et al., 2014a). However, no clear evidence of H3K27me3 marks on OR genes has been found yet (Magklara et al., 2011), although Armelin-Correa et al. (2014a) report H3K27me3 markings being required for asymmetric replication of OR genes in embryonic stem cells.

Recent studies show that early developing OSNs can weakly express multiple OR genes, while during subsequent stages of development the expression of one single OR gene overtakes and the other OR *loci* get silenced (Hanchate et al., 2015; Saraiva et al., 2015; Tan et al., 2015; Scholz et al., 2016). To explain this transition, Hanchate et al. (2015) proposed a *winner takes all*-model where one of the initially expressed OR genes becomes dominant, capturing limiting factors required for high expression level. Alternatively, the high expression of one OR gene would occur independently of other earlier expressed genes. Hanchate et al. (2015) also suggest a *regional bias* in OR gene choice: early co-expressed OR genes, although sitting at multiple chromosomal locations, are expressed in neurons located in the same region of MOE.

Immature OSNs expressing an OR gene can still switch to another OR gene in a loop-process that continues until a functional OR gene is expressed and elicits a feedback signal that stops the cycle and stabilizes the choice (Serizawa et al., 2003; Lewcock and Reed, 2004; Shykind et al., 2004). In post-mitotic OSNs, a single OR gene – chosen in a stochastic, yet elusive event – escapes *foci* and gets repositioned in a nearby nuclear area (Clowney et al., 2012). According to Lyons et al. (2013) a derepressor with limited availability, either in space or time, would act together with the histone lysine demethylase 1 (Lsd1), transiently expressed at the core time window of OR gene choice. This event is associated with an epigenetic switch from H3K9me3 to H3K4me3 for the chosen OR allele, which perhaps interacts with an interchromosomal complex of elements (Magklara et al., 2011; Lyons et al., 2013; Markenscoff-Papadimitriou et al., 2014). Currently, it is unclear whether H3K27 demethylases may have a role in the process too (Armelin-Correa et al., 2014a).

**FIGURE 1:**
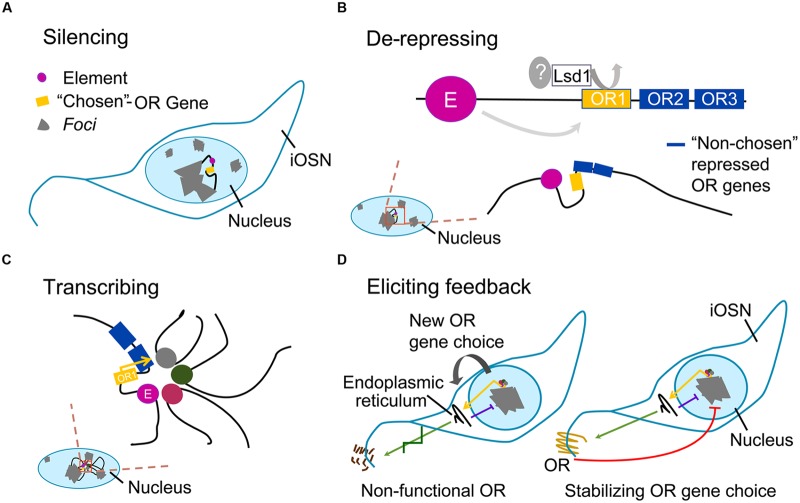
**Main steps of odorant receptor gene choice.** Gray areas represent *foci;* black filament represents euchromatin; colored circles represent elements; yellow box represents the single –“chosen” odorant receptor (OR) allele; blue boxes represent nearby OR genes with repressive marks. **(A)** Silencing: in the nucleus of maturing olfactory sensory neuron (iOSN), OR gene *loci* are heterochromatized; one *locus* undergoes an epigenetic change. **(B)** De-repressing: local variation in epigenetic state on OR heterochromatin (magnified shadowed red-stroked box) is initiated by an unknown derepressor, which cooperates with Lsd1 and perhaps with H3K27 demethylases in the random opening of one OR allele only; an element in the same OR *locus* interacts with the OR gene via DNA-looping; nearby OR genes keep their repressive marks. **(C)** Transcribing: on the euchromatic OR allele, an interchromosomal complex of elements drives robust expression of the gene. **(D)** Eliciting feedback: if massive protein production within the endoplasmic reticulum is achieved, unfolded protein response is triggered; this causes Adcy3-mediated block on Lsd1, resulting in cell inability to unpack silenced OR *loci* and to re-close the euchromatized allele (purple line); *left*, if the “chosen-OR” is a pseudogene the process is repeated with a new OR-choice; *right*, if a functional OR protein is produced (green arrow), its activity leads to the release of the βγ subunit of the G protein, which further prevents other OR alleles to escape *foci* (red line); therefore, in order to stabilize OR gene choice, the process induces odorant sensory neuron (OSN) maturation.

The expression of an intact OR gene activates the unfolded protein response, which eventually leads to the production of adenylate cyclase 3 (Adcy3); Adcy3 represses Lsd1 and promotes neuronal maturation, locking OR gene choice. If the OR gene is nonfunctional and fails to elicit Adcy3-mediated feedback, Lsd1 retains its activity: it might re-heterochromatize the opened *locus*, or alternatively it may open another one. This process of choice (**Figure 1**) would continue until an OR gene succeeds in being stably expressed (Dalton et al., 2013).

Whereas developmental expression seems to be independent from odorant receptor-induced neuronal activity (Hanchate et al., 2015), Ferreira et al. (2014) have shown in zebrafish that the βγ subunit of the olfactory G protein, released when an OR binds its ligand, has a direct impact on the methylation state of silenced OR *loci*, thus linking receptor activity to the epigenetic regulation behind the single OR gene choice mechanism.

More recently Zhang et al. (2016) have shown that the homeodomain transcription factor Lhx2 influences OR expression frequencies in immature and mature OSNs, and it is necessary for driving OR expression but not for the OR singularity, although they do not exclude an indirect role in OR gene choice.

## Concluding Remarks

Overall, OR genes seem to adopt a lock-and-key strategy for expression; all *loci* are initially epigenetically silenced, then a limiting factor randomly opens a single allele that later on stabilizes its own transcription through complex feedback mechanisms. Aside from the olfactory system, others examples of way to increase cellular diversity among similar cell types are provided by immune system (Hozumi and Tonegawa, 1976; Jaeger et al., 2013; Magklara and Lomvardas, 2013), and protocadherins (Lefebvre et al., 2012). Several transcriptional characteristics seem to recur in other clustered gene families, such as globins and homeobox genes, which also display oligogenic expression. However, whilst globins (Drescher and Künzer, 1954; Huehns et al., 1964; Groudine et al., 1983) and homeobox (Gaunt et al., 1988; Duboule and Dollé, 1989; Dressler and Gruss, 1989; Graham et al., 1989) genes are serially expressed according to their chromosomal location, OR gene family requires more complex regulation. What are the molecular mechanisms that lead to the OR gene expression? How is the OSN transition to a single highly expressed OR gene regulated? How does the nuclear architecture influence this process? What is the missing link between OR gene expression and the mature OSN identity? These are only few of the fundamental open questions still tickling the olfaction field.

## Author Contributions

ADI drafted an early version of the manuscript, AD critically revised it. ADI and AD wrote the manuscript. All authors read and accepted the final version.

## Conflict of Interest Statement

The authors declare that the research was conducted in the absence of any commercial or financial relationships that could be construed as a potential conflict of interest.

## References

[B1] Armelin-Correa L. M. Gutiyama L. M. Brandt D. Y. Malnic B. (2014a). Nuclear compartmentalization of odorant receptor genes. *Proc. Natl. Acad. Sci. U.S.A.* 111 2782–2787. 10.1073/pnas.1317036111 24550308 PMC3932893

[B2] Armelin-Correa L. M. Nagai M. H. Leme Silva A. G. Malnic B. (2014b). Nuclear architecture and gene silencing in olfactory sensory neurons. *Bioarchitecture* 4 160–163. 10.4161/19490992.2014.982934 25714005 PMC4914026

[B3] Bozza T. Vassalli A. Fuss S. Zhang J. J. Weiland B. Pacifico R. (2009). Mapping of class I and class II odorant receptors to glomerular domains by two distinct types of olfactory sensory neurons in the mouse. *Neuron* 61 220–233. 10.1016/j.neuron.2008.11.010 19186165 PMC3013286

[B4] Breer H. Fleischer J. Strotmann J. (2006). The sense of smell: multiple olfactory subsystems. *Cell. Mol. Life Sci.* 63 1465–1475. 10.1007/s00018-006-6108-5 16732429 PMC11136015

[B5] Buck L. Axel R. (1991). A novel multigene family may encode odorant receptors: a molecular basis for odor recognition. *Cell* 65 175–187. 10.1016/0092-8674(91)90418-X 1840504

[B6] Bufe B. Schumann T. Kappl R. Bogeski I. Kummerow C. Podgorska M. (2015). Recognition of bacterial signal peptides by mammalian formyl peptide receptors. *J. Biol. Chem.* 290 7369–7387. 10.1074/jbc.M114.626747 25605714 PMC4367248

[B7] Chess A. Simon I. Cedar H. Axel R. (1994). Allelic inactivation regulates olfactory receptor gene expression. *Cell* 78 823–834. 10.1016/S0092-8674(94)90562-2 8087849

[B8] Clowney E. J. Legros M. A. Mosley C. P. Clowney F. G. Markenskoff-Papadimitriou E. C. Myllys M. (2012). Nuclear aggregation of olfactory receptor genes governs their monogenic expression. *Cell* 151 724–737. 10.1016/j.cell.2012.09.043 23141535 PMC3659163

[B9] Clowney E. J. Magklara A. Colquitt B. M. Pathak N. Lane R. P. Lomvardas S. (2011). High-throughput mapping of the promoters of the mouse olfactory receptor genes reveals a new type of mammalian promoter and provides insight into olfactory teceptor gene regulation. *Genome Res.* 21 1249–1259. 10.1101/gr.120162.110 21705439 PMC3149492

[B10] Colquitt B. M. Markenscoff-Papadimitriou E. Duffié R. Lomvardas S. (2014). Dnmt3a regulates global gene expression in olfactory sensory neurons and enables odorant-induced transcription. *Neuron* 83 823–838. 10.1016/j.neuron.2014.07.013 25123312 PMC4153871

[B11] Dalton R. P. Lyons D. B. Lomvardas S. (2013). Co-opting the unfolded protein response to elicit olfactory receptor feedback. *Cell* 155 321–332. 10.1016/j.cell.2013.09.033 24120133 PMC3843319

[B12] Degl’Innocenti A. Parrilla M. Harr B. Teschke M. (2016). The mouse solitary odorant receptor gene promoters as models for the study of odorant receptor gene choice. *PLoS ONE* 11:e0144698 10.1371/journal.pone.0144698 PMC4721658 26794459

[B13] Døving K. B. Trotier D. (1998). Structure and function of the vomeronasal organ. *J. Exp. Biol.* 201(Pt 21), 2913–2925. 9866877 10.1242/jeb.201.21.2913

[B14] Drescher H. Künzer W. (1954). Der Blutfarbstoff des menschlichen Feten. *Klin. Wochenschr.* 32 92 10.1007/bf01493536 13143695

[B15] Dressler G. R. Gruss P. (1989). Anterior boundaries of hox gene expression in mesoderm-derived structures correlate with the linear gene order along the chromosome. *Differentiation* 41 193–201. 10.1111/j.1432-0436.1989.tb00747.x 2575552

[B16] Duboule D. Dollé P. (1989). The structural and functional organization of the murine HOX gene family resembles that of drosophila homeotic genes. *EMBO J.* 8 1497–1505. 2569969 10.1002/j.1460-2075.1989.tb03534.x PMC400980

[B17] Dulac C. Axel R. (1995). A novel family of genes encoding putative pheromone receptors in mammals. *Cell* 83 195–206. 10.1016/0092-8674(95)90161-2 7585937

[B18] Eggan K. Baldwin K. Tackett M. Osborne J. Gogos J. Chess A. (2004). Mice cloned from olfactory sensory neurons. *Nature* 428 44–49. 10.1038/nature02375 14990966

[B19] Enomoto T. Ohomoto M. Iwata T. Uno T. Saitou M. Yamaguchi T. (2011). Bcl11b/Ctip2 controls the differentiation of vomeronasal sensory neurons in mice. *J. Neurosci.* 31 10159–10173. 10.1523/JNEUROSCI.1245-11.2011 21752992 PMC3394408

[B20] Ferreira T. Wilson S. R. Choi Y. G. Risso D. Dudoit S. Speed T. P. (2014). Silencing of odorant receptor genes by G protein βγ signaling ensures the expression of one odorant receptor per olfactory sensory neuron. *Neuron* 81 847–859. 10.1016/j.neuron.2014.01.001 24559675 PMC4412037

[B21] Fleischer J. Breer H. (2010). The grueneberg ganglion: a novel sensory system in the nose. *Histol. Histopathol.* 25 909–915. 20503179 10.14670/HH-25.909

[B22] Gaunt S. J. Sharpe P. T. Duboule D. (1988). Spatially restricted domains of homeo-gene transcripts in mouse embryos: relation to a segmented body plan. *Development* 104(Suppl.), 169–179.

[B23] Godfrey P. A. Malnic B. Buck L. B. (2004). The mouse olfactory receptor gene family. *Proc. Natl. Acad. Sci. U.S.A.* 17 2156–2161. 10.1073/pnas.0308051100 PMC357068 14769939

[B24] Graham A. Papalopulu N. Krumlauf R. (1989). The murine and drosophila homeobox gene complexes have common features of organization and expression. *Cell* 57 367–378. 10.1016/0092-8674(89)90912-4 2566383

[B25] Greer P. L. Bear D. M. Lassance J. Bloom M. L. Tsukahara T. Pashkovski S. L. (2016). A family of non-GPCR chemosensors defines an alternative logic for mammalian olfaction. *Cell* 165 1734–1748. 10.1016/j.cell.2016.05.001 27238024 PMC4912422

[B26] Groudine M. Kohwi-Shigematsu T. Gelinas R. Stamatoyannopoulos G. Papayannopoulou T. (1983). Human fetal to adult hemoglobin switching: changes in chromatin structure of the beta-globin gene locus. *Proc. Natl. Acad. Sci. U.S.A.* 80 7551–7555. 10.1073/pnas.80.24.7551 6200877 PMC534378

[B27] Halpern M. (1987). The organization and the function of the vomeronasal system. *Ann. Rev. Neurosci.* 10 325–362. 10.1146/annurev.ne.10.030187.001545 3032065

[B28] Hanchate N. K. Kondoh K. Lu Z. Kuang D. Ye X. Qiu X. (2015). Single-cell transcriptomics reveals receptor transformations during olfactory neurogenesis. *Science* 3 123–128. 10.1126/science.aad2456 PMC5642900 26541607

[B29] Herrada G. Dulac C. (1997). A novel family of putative pheromone receptors in mammals with a topographically organized and sexually dimorphic distribution. *Cell* 90 763–773. 10.1016/S0092-8674(00)80536-X 9288755

[B30] Hirota J. Mombaerts P. (2004). The LIM-homeodomain protein Lhx2 is required for complete development of mouse olfactory sensory neurons. *Proc. Natl. Acad. Sci. U.S.A.* 101 8751–8755. 10.1073/pnas.0400940101 15173589 PMC423267

[B31] Hirota J. Omura M. Mombaerts P. (2007). Differential impact of Lhx2 deficiency on expression of class I and class II odorant receptor genes in mouse. *Mol. Cell. Neurosci.* 34 679–688. 10.1016/j.mcn.2007.01.014 17350283

[B32] Hoppe R. Breer H. Strotmann J. (2006). Promoter motifs of olfactory receptor genes expressed in distinct topographic patterns. *Genomics* 87 711–723. 10.1016/j.ygeno.2006.02.005 16600568

[B33] Hoppe R. Frank H. Breer H. Strotmann J. (2003). The clustered olfactory receptor gene family 262: genomic organization, promotor elements, and interacting transcription factors. *Genome Res.* 13 2674–2685. 10.1101/gr.1372203 14656972 PMC403809

[B34] Hozumi N. Tonegawa S. (1976). Evidence for somatic rearrangement of immunoglobulin genes coding for variable and constant regions. *Proc. Natl. Acad. Sci. U.S.A.* 73 3628–3632. 10.1073/pnas.73.10.3628 824647 PMC431171

[B35] Huehns E. R. Dance N. Beaven G. H. Keil J. V. Hecht F. Motulsky A. G. (1964). Human embryonic haemoglobins. *Nature* 14 1095–1097. 10.1038/2011095a0 14152781

[B36] Ibarra-Soria X. Levitin M. O. Saraiva L. R. Logan D. W. (2014). The olfactory transcriptomes of mice. *PLoS Genet.* 10:e1004593 10.1371/journal.pgen.1004593 PMC4154679 25187969

[B37] Ishii T. Mombaerts P. (2008). Expression of nonclassical class I major histocompatibility genes defines a tripartite organization of the mouse vomeronasal system. *J. Neurosci.* 28 2332–2341. 10.1523/JNEUROSCI.4807-07.2008 18322080 PMC6671199

[B38] Ishii T. Mombaerts P. (2011). Coordinated coexpression of two vomeronasal receptor V2R genes per neuron in the mouse. *Mol. Cell. Neurosci.* 46 397–408. 10.1016/j.mcn.2010.11.002 21112400

[B39] Jaeger S. Fernandez B. Ferrier P. (2013). Epigenetic aspects of lymphocyte antigen receptor gene rearrangement or ‘when stochasticity completes randomness’. *Immunology* 139 141–150. 10.1111/imm.12057 23278765 PMC3647179

[B40] Jia C. Halpern M. (1996). Subclasses of vomeronasal receptor neurons: differential expression of G proteins (Gi alpha 2 and G(O alpha)) and segregated projections to the accessory olfactory bulb. *Brain Res.* 719 117–128. 10.1016/0006-8993(96)00110-2 8782871

[B41] Jiang Y. Gong N. N. Hu X. S. Ni M. J. Pasi R. Matsunami H. (2015). Molecular profiling of activated olfactory neurons identifies odorant receptors for odors in vivo. *Nat. Neurosci.* 18 1446–1454. 10.1038/nn.4104 26322927 PMC4583814

[B42] Kanageswaran N. Demond M. Nagel M. Schreiner B. S. Baumgart S. Scholz P. (2015). Deep sequencing of the murine olfactory receptor neuron transcriptome. *PLoS ONE* 10:e0113170 10.1371/journal.pone.0113170 PMC4295871 25590618

[B43] Khan M. Vaes E. Mombaerts P. (2011). Regulation of the probability of mouse odorant receptor gene choice. *Cell* 147 907–921. 10.1016/j.cell.2011.09.049 22078886

[B44] Lane R. P. Cutforth T. Young J. Athanasiou M. Friedman C. Rowen L. (2001). Genomic analysis of ortholo- gous mouse and human olfactory receptor loci. *Proc. Natl. Acad. Sci. U.S.A.* 98 7390–7395. 10.1073/pnas.131215398 11416212 PMC34679

[B45] Le Gros Clark W. E. Turner Warwick R. T. (1946). The pattern of olfactory innervation. *J. Neurol. Neurosurg. Psychiatry* 9 101–111. 10.1136/jnnp.9.3.101 20293592 PMC497093

[B46] Lefebvre J. L. Kostadinov D. Chen W. V. Maniatis T. Sanes J. R. (2012). Protocadherins mediate dendritic self-avoidance in the mammalian nervous system. *Nature* 488 517–521. 10.1038/nature11305 22842903 PMC3427422

[B47] Leinders-Zufall T. Ishii T. Mombaerts P. Zufall F. Boehm T. (2009). Structural requirements for the activation of vomeronasal sensory neurons by MHC peptides. *Nat. Neurosci.* 12 1551–1558. 10.1038/nn.2452 19935653

[B48] Lewcock J. W. Reed R. R. (2004). A feedback mechanism regulates monoallelic odorant receptor expression. *Proc. Natl. Acad. Sci. U.S.A.* 101 1069–1074. 10.1073/pnas.0307986100 14732684 PMC327152

[B49] Li J. Ishii T. Feinstein P. Mombaerts P. (2004). Odorant receptor gene choice is reset by nuclear transfer from mouse olfactory sensory neurons. *Nature* 428 393–399. 10.1038/nature02433 15042081

[B50] Liberles S. D. Buck L. B. (2006). A second class of chemosensory receptors in the olfactory epithelium. *Nature* 442 645–650. 10.1038/nature05066 16878137

[B51] Lomvardas S. Barnea G. Pisapia D. J. Mendelsohn M. Kirkland J. Axel R. (2006). Interchromosomal interactions and olfactory receptor choice. *Cell* 126 403–413. 10.1016/j.cell.2006.06.035 16873069

[B52] Lyons D. B. Allen W. E. Goh T. Tsai L. Barnea G. Lomvardas S. (2013). An epigenetic trap stabilizes singular olfactory receptor expression. *Cell* 154 325–336. 10.1016/j.cell.2013.06.039 23870122 PMC3929589

[B53] Magklara A. Lomvardas S. (2013). Stochastic gene expression in mammals: lessons from olfaction. *Trends Cell Biol.* 23 449–456. 10.1016/j.tcb.2013.04.005 23689023 PMC3755038

[B54] Magklara A. Yen A. Colquitt B. M. Clowney E. J. Allen W. Markenscoff-Papadimitriou E. (2011). An epigenetic signature for monoallelic olfactory receptor expression. *Cell* 145 555–570. 10.1016/j.cell.2011.03.040 21529909 PMC3094500

[B55] Malnic B. Godfrey P. A. Buck L. B. (2004). The human olfactory receptor gene family. *Proc. Natl. Acad. Sci. U.S.A.* 101 2584–2589. 10.1073/pnas.0307882100 14983052 PMC356993

[B56] Markenscoff-Papadimitriou E. Allen W. E. Colquitt B. M. Goh T. Murphy K. K. Monahan K. (2014). Enhancer interaction networks as a means for singular olfactory receptor expression. *Cell* 159 543–557. 10.1016/j.cell.2014.09.033 25417106 PMC4243057

[B57] Martini S. Silvotti L. Shirazi A. Ryba N. J. Tirindelli R. (2001). Co-expression of putative pheromone receptors in the sensory neurons of the vomeronasal organ. *J. Neurosci.* 21 843–848. 11157070 10.1523/JNEUROSCI.21-03-00843.2001 PMC6762303

[B58] Matsunami H. Buck L. B. (1997). A multigene family encoding a diverse array of putative pheromone receptors in mammals. *Cell* 90 775–784. 10.1016/S0092-8674(00)80537-1 9288756

[B59] McCotter R. E. (1912). The connection of the vomeronasal nerves with the accessory olfactory bulb in the opossum and other mammals. *Anat. Rec.* 6 299–318. 10.1002/ar.1090060802

[B60] Mendoza A. S. (1993). Morphological studies on the rodent main and accessory olfactory systems: the regio olfactoria and vomeronasal organ. *Ann. Anat.* 175 425–446. 10.1016/S0940-9602(11)80110-X 8250272

[B61] Michaloski J. S. Galante P. A. Nagai M. H. Armelin-Correa L. Chien M. S. Matsunami H. (2011). Common promoter elements in odorant and vomeronasal receptor genes. *PLoS ONE* 6:e29065 10.1371/journal.pone.0029065 PMC3247230 22216168

[B62] Michaloski J. S. Galante P. A. F. Malnic B. (2006). Identification of potential regulatory motifs in odorant receptor genes by analysis of promoter sequences. *Genome Res.* 16 1091–1098. 10.1101/gr.5185406 16902085 PMC1557771

[B63] Mombaerts P. Wang F. Dulac C. Chao S. K. Nemes A. Mendelsohn M. (1996). Visualizing an olfactory sensory map. *Cell* 87 675–686. 10.1016/S0092-8674(00)81387-2 8929536

[B64] Movahedi K. Grosmaitre X. Finestein P. (2016). Odorant receptors can mediate axonal identity and gene choice via cAMP-indipendent mechanisms. *Open Biol.* 6 160018 10.1098/rsob.160018 PMC4967819 27466441

[B65] Nara K. Saraiva L. R. Ye X. Buck L. B. (2011). A large-scale analysis of odor coding in the olfactory epithelium. *J. Neurosci.* 31 9179–9191. 10.1523/JNEUROSCI.1282-11.2011 21697369 PMC3758579

[B66] Ngai J. Chess A. Dowling M. M. Necles N. Macagno E. R. Axel R. (1993a). Coding of olfactory information: topography of odorant receptor expression in the catfish olfactory epithelium. *Cell* 72 667–680. 8453662 10.1016/0092-8674(93)90396-8

[B67] Ngai J. Dowling M. M. Buck L. Axel R. Chess A. (1993b). The family of genes encoding odorant receptors in the channel catfish. *Cell* 72 657–666. 7916654 10.1016/0092-8674(93)90395-7

[B68] Niimura Y. Matsui A. Touhara K. (2014). Extreme expansion of the olfactory receptor gene repertoire in african elephants and evolutionary dynamics of orthologous gene groups in 13 placental mammals. *Genome Res.* 24 1485–1496. 10.1101/gr.169532.113 25053675 PMC4158756

[B69] Nishizumi H. Kumasaka K. Inoue N. Nakashima A. Sakano H. (2007). Deletion of the core-H region in mice abolishes the expression of three proximal odorant receptor genes in cis. *Proc. Natl. Acad. Sci. U.S.A.* 104 20067–20072. 10.1073/pnas.0706544105 18077433 PMC2148423

[B70] Plessy C. Pascarella G. Bertin N. Akalin A. Carrieri C. Vassalli A. (2012). Promoter architecture of mouse olfactory receptor genes. *Genome Res.* 22 486–497. 10.1101/gr.126201.111 22194471 PMC3290784

[B71] Ressler K. J. Sullivan S. L. Buck L. (1994). Information coding in the olfactory system: evidence for a stereotyped and highly organized epitope map in the olfactory bulb. *Cell* 79 1245–1255. 10.1016/0092-8674(94)90015-9 7528109

[B72] Rivière S. Challet L. Fluegge D. Spehr M. Rodriguez I. (2009). Formil peptide receptor-like proteins are a novel family of vomeronasal chemosensors. *Nature* 459 574–577. 10.1038/nature08029 19387439

[B73] Rodriguez I. Del Punta K. Rothman A. Ishii T. Mombaerts P. (2002). Multiple new and isolated families within the mouse superfamily of V1r vomeronasal receptors. *Nat. Neurosci.* 5 134–140. 10.1038/nn795 11802169

[B74] Rodriguez I. Feinstein P. Mombaerts P. (1999). Variable patterns of axonal projections of sensory neurons in the mouse vomeronasal system. *Cell* 97 199–208. 10.1016/S0092-8674(00)80730-8 10219241

[B75] Roppolo D. Vollery S. Kan C. D. Lüscher C. Broillet M. C. Rodriguez I. (2007). Gene cluster lock after pheromone receptor gene choice. *EMBO J.* 26 3423–3430. 10.1038/sj.emboj.7601782 17611603 PMC1933412

[B76] Rothman A. Feinstein P. Hirota J. Mombaerts P. (2005). The promoter of the mouse odorant receptor gene M71. *Mol. Cell. Neurosci.* 28 535–546. 10.1016/j.mcn.2004.11.006 15737743

[B77] Ryba N. J. Tirindelli R. (1997). A new multigene family of putative pheromone receptors. *Neuron* 19 371–379. 10.1016/S0896-6273(00)80946-0 9292726

[B78] Santoro S. W. Dulac C. (2012). The activity-dependent histone variant H2BE modulates the life span of olfactory neurons. *Elife* 13:e00070 10.7554/eLife.00070 PMC3510456 23240083

[B79] Saraiva L. R. Ibarra-Soria X. Khan M. Omura M. Scialdone A. Mombaerts P. (2015). Hierarchical deconstruction of mouse olfactory sensory neurons: from whole mucosa to single-cell RNA-seq. *Sci. Rep.* 5:18178 10.1038/srep18178 PMC4680959 26670777

[B80] Scholz P. Kalbe B. Jansen F. Altmueller J. Becker C. Mohrhardt J. (2016). Transcriptome analysis of murine olfactory sensory neurons during development using single cell RNA-Seq. *Chem. Senses* 41 313–323. 10.1093/chemse/bjw003 26839357

[B81] Serizawa S. Ishii T. Nakatani H. Tsuboi A. Nagawa F. Asano M. (2000). Mutually exclusive expression of odorant receptor transgenes. *Nat. Neurosci.* 3 687–693. 10.1038/76641 10862701

[B82] Serizawa S. Miyamichi K. Nakatani H. Suzuki M. Saito M. Yoshihara Y. (2003). Negative feedback regulation ensures the one receptor-one olfactory neuron rule in mouse. *Science* 302 2088–2094. 10.1126/science.1089122 14593185

[B83] Shykind B. M. Rohani S. C. O’Donnell S. Nemes A. Mendelsohn M. Sun Y. (2004). Gene switching and the stability of odorant receptor gene choice. *Cell* 117 801–815. 10.1016/j.cell.2004.05.015 15186780

[B84] Silvotti L. Moiani A. Gatti R. Tirindelli R. (2007). Combinatorial co-expression of pheromone receptors, V2Rs. *J. Neurochem.* 103 1753–1763. 10.1111/j.1471-4159.2007.04877.x 17854397

[B85] Solovei I. Kreysing M. Lanctôt C. Kösem S. Peichl L. Cremer T. (2009). Nuclear architecture of rod photoreceptor cells adapts to vision in mammalian evolution. *Cell* 137 356–368. 10.1016/j.cell.2009.01.052 19379699

[B86] Sullivan S. L. Adamson M. C. Ressler K. J. Kozak C. A. Buck L. B. (1996). The chromosomal distribution of mouse odorant receptor genes. *Proc. Natl. Acad. Sci. U.S.A.* 93 884–888. 10.1073/pnas.93.2.884 8570653 PMC40152

[B87] Tan L. Li Q. Xie S. (2015). Olfactory sensory neurons transiently express multiple olfactory receptors during development. *Mol. Syst. Biol.* 11:844 10.15252/msb.20156639 PMC4704490 26646940

[B88] Tian X. J. Zhang H. Sannerud J. Xing J. (2016). Achieving diverse and monoallelic olfactory receptor selection through dual-objective optimization design. *Proc. Natl. Acad. Sci. U.S.A.* 113 E2889–E2898. 10.1073/pnas.1601722113 27162367 PMC4889386

[B89] Tirindelli R. Dibattista M. Pifferi S. Menini A. (2009). From pheromones to behavior. *Physiol. Rev.* 89 921–956. 10.1152/physrev.00037.2008 19584317

[B90] Vassalli A. Feinstein P. Mombaerts P. (2011). Homeodomain binding motifs modulate the probability of odorant receptor gene choice in transgenic mice. *Mol. Cell. Neurosci.* 46 381–396. 10.1016/j.mcn.2010.11.001 21111823 PMC3746036

[B91] Vassalli A. Rothman A. Feinstein P. Zapotocky M. Mombaerts P. (2002). Minigenes impart odorant receptor-specific axon guidance in the olfactory bulb. *Neuron* 35 681–696. 10.1016/S0896-6273(02)00793-6 12194868

[B92] Vassar R. Chao S. K. Sitcheran R. Nuñez J. M. Vosshall L. B. Axel R. (1994). Topographic organization of sensory projections to the olfactory bulb. *Cell* 79 981–991. 10.1016/0092-8674(94)90029-9 8001145

[B93] Wang F. Nemes A. Mendelsohn M. Axel R. (1998). Odorant receptors govern the formation of a precise topographic map. *Cell* 93 47–60. 10.1016/S0092-8674(00)81145-9 9546391

[B94] Wang S. S. Tsai R. Y. Reed R. R. (1997). The characterization of the Olf-1/EBF-like HLH transcription factor family: implications in olfactory gene regulation and neuronal development. *J. Neurosci.* 17 4149–4158. 9151732 10.1523/JNEUROSCI.17-11-04149.1997 PMC6573563

[B95] Young J. M. Friedman C. Williams E. M. Ross J. A. Tonnes-Priddy L. Trask B. J. (2002). Different evolutionary processes shaped the mouse and human olfactory receptor gene families. *Hum. Mol. Genet.* 11 535–546. Erratum in: *Hum. Mol. Genet.* 11:1683 10.1093/hmg/11.5.535 11875048

[B96] Young J. M. Luche R. M. Trask B. J. (2011). Rigorous and thorough bioinformatic analyses of olfactory receptor promoters confirm enrichment of O/E and homeodomain binding sites but reveal no new common motifs. *BMC Genomics* 12:561 10.1186/1471-2164-12-561 PMC3247239 22085861

[B97] Zhang G. Titlow W. B. Biecker S. M. Stromberg A. J. McClintock T. S. (2016). Lhx2 determines odorant receptor expression frequency in mature olfactory sensory neurons. *eNeuro* 3 10.1523/eneuro.0230-16.2016 PMC5086798 27822500

[B98] Zhang X. Firestein S. (2002). The olfactory receptor gene superfamily of the mouse. *Nat. Neurosci.* 5 124–133. 11802173 10.1038/nn800

[B99] Zhang X. Rodriguez I. Mombaerts P. Firestein S. (2004). Odorant and vomeronasal receptor genes in two mouse genome assemblies. *Genomics* 83 802–811. 10.1016/j.ygeno.2003.10.009 15081110

[B100] Zhang X. Zhang X. Firestein S. (2007). Comparative genomics of odorant- and pheromone receptor genes in rodents. *Genomics* 89 441–450. 10.1016/j.ygeno.2007.01.002 17303377 PMC1992438

